# Advances in 3D-Printed Drug Delivery and Screening Platforms for Bone Disease Therapy

**DOI:** 10.3390/pharmaceutics17111372

**Published:** 2025-10-23

**Authors:** Iosif-Aliodor Timofticiuc, Alex-Gabriel Grigore, Elena-Teodora Tomescu, Teona-Maria Vlaicu, Serban Dragosloveanu, Andreea-Elena Scheau, Ana Caruntu, Christiana Diana Maria Dragosloveanu, Ioana Anca Badarau, Andreea Cristiana Didilescu, Constantin Caruntu, Cristian Scheau

**Affiliations:** 1 Faculty of Medicine, The “Carol Davila” University of Medicine and Pharmacy, 8 Eroii Sanitari Boulevard, 050474 Bucharest, Romania; 2 “Foisor” Clinical Hospital of Orthopedics, Traumatology and Osteoarticular TB, 021382 Bucharest, Romania; 3 Department of Orthopedics and Traumatology, The “Carol Davila” University of Medicine and Pharmacy, 050474 Bucharest, Romania; 4 Department of Orthopedics, “Foisor” Clinical Hospital of Orthopedics, Traumatology and Osteoarticular TB, 021382 Bucharest, Romania; 5 Department of Radiology and Medical Imaging, “Foisor” Clinical Hospital of Orthopedics, Traumatology and Osteoarticular TB, 021382 Bucharest, Romania; 6 Department of Oral and Maxillofacial Surgery, “Carol Davila” Central Military Emergency Hospital, 010825 Bucharest, Romania; 7 Faculty of Dental Medicine, “Titu Maiorescu” University, 031593 Bucharest, Romania; 8 Department of Ophthalmology, Faculty of Dentistry, The “Carol Davila” University of Medicine and Pharmacy, 050474 Bucharest, Romania; 9 Department of Ophthalmology, Clinical Hospital for Ophthalmological Emergencies, 010464 Bucharest, Romania; 10 Department of Physiology, The “Carol Davila” University of Medicine and Pharmacy, 8 Eroii Sanitari Boulevard, 050474 Bucharest, Romania; 11 Department of Embryology and Microbiology, Faculty of Dentistry, The “Carol Davila” University of Medicine and Pharmacy, 050474 Bucharest, Romania; 12 Department of Dermatology, “Prof. N.C. Paulescu,” National Institute of Diabetes, Nutrition and Metabolic Diseases, 011233 Bucharest, Romania

**Keywords:** 3D printing, drug delivery, drug screening, bone diseases, patient-specific, orthopedics, physiopathology

## Abstract

Bone diseases such as osteomyelitis, osteosarcoma, and osteoarthritis, as well as conditions caused by metabolic imbalances, including osteoporosis, require more efficient and optimized therapies. Systemic drug administration entails major disadvantages like cytotoxicity and adverse reactions, which can lead to serious complications or death. Therefore, local drug administration alternatives are currently under investigation for different pharmacological therapies. New vectors were created to improve control over administration, and 3D-printed and patient-specific drug delivery systems have been tested, revealing great potential. Moreover, 3D-printed platforms that mimic human tissues for drug testing are innovative solutions emerging for the pharmaceutical industry. Situated between in vitro and in vivo testing on human patients, they offer the advantage of reproducing functional architecture, providing results that are closer to those encountered in clinical trials performed on patients. In our article, we present the two categories of 3D systems, from the perspective of main drug groups (antibiotics, anticancer, and anti-inflammatory) as well as other categories, alongside their advantages, limitations, and their adaptations to 3D printing technologies. This article also highlights the technological drawbacks encountered in both delivery and screening systems, as well as the printing methods and materials used, including their physical and biological properties.

## 1. Introduction

Three-dimensional (3D) printing has emerged in recent decades as one of the most promising and potent tools to fabricate complex, personalized products on demand with relative ease and low costs [1]. The technology has been widely used and implemented in a multitude of sectors, including automotive, construction, aerospace, medical, and many other industries [2,3,4]. In the pharmaceutical sector, 3D printing has enabled the development of personalized drug delivery systems, such as patient-specific tablets with controlled release profiles [5,6]. It also facilitates the rapid prototyping of medical devices and tissue engineering scaffolds [7]. Furthermore, this technology supports the fabrication of advanced screening platforms for disease modulation and targeted drug response, allowing for high-throughput, physiologically relevant testing [8,9,10,11]. Its precise control over formulation architecture further enhances the study of drug release kinetics and biological responses in preclinical models.

Due to these advantages of 3D printing, this technology has been especially significant in the field of orthopedics and craniofacial surgery, particularly in bone pathologies where traditional models may not sufficiently capture the complexity or biology of bone tissue architecture [12]. Bone-specific diseases, including osteoporosis, osteoarthritis, and bone cancers, pose unique therapeutic and modeling challenges to the development of therapeutics, drugs, and biologics because of the complexities of their pathophysiology and the need for a targeted, localized treatment approach [13,14,15]. Typically, traditional techniques do not allow for drugs to be effectively delivered to the bone or lack the ability to replicate the native bone microenvironment for testing. 3D printing has opened doors for designing and fabricating bone-mimetic scaffolds, as well as in vitro models of bone diseases that would exhibit structural and mechanical properties of native bone [16,17]. The ability to control the architecture and composition of a material enhances the application of 3D printing in studying more physiologically relevant conditions, including bone regeneration processes, drug release characteristics, and cellular behavior.

Regarding bone diseases, drug delivery systems are defined as specialized formulations or devices designed to bring and release therapeutic agents to the affected tissue [18]. The use of traditional systemic drug delivery frequently results in insufficient drug concentrations at the bone site with reduced efficacy, and an increased risk for systemic toxicity, especially when drug efficacy is limited by extensive bone damage [19]. In this case, being able to deliver an appropriate concentration locally at the site of disease is fundamental for achieving the desired bone concentrations, while limiting systemic drug levels and unintended side effects [20,21,22]. There are numerous examples of materials used to create effective bone drug delivery systems, including, but not limited to, biodegradable polymers (e.g., PLGA, PCL) [23,24], lipids (i.e., phospholipids in liposomes) [21,25], inorganic agents (e.g., silica, calcium phosphate) [26,27], and hydrogels [28]. Each has a range of benefits, including controlled release, targeting, and biocompatibility in therapeutic applications. Additionally, extensive research is conducted on using advanced materials such as metal-organic frameworks (MOFs) [29] and bioactive ceramics [30] for specialized applications like cancer therapy or for sustaining bone regeneration. Drug screening platforms, however, may be defined as in vitro or ex vivo models that mimic the tissue microenvironment and are useful in the assessment of the efficacy and safety of potential treatments [31,32]. Traditional models may be physiologically irrelevant due to the system‘s simplistic architecture and failure to allow dynamic interactions [33]. Advanced 3D bone mimetic platforms are attempting to provide structural, mechanical, and cellular complexity similar to native bone, in order to permit more predictive in vitro testing [34]. These systems are oriented towards addressing the limitations of traditional models, which did not allow for close cell–cell and cell–matrix interactions while also enabling high-throughput screening and real-time monitoring [33,35].

**Figure 1:**
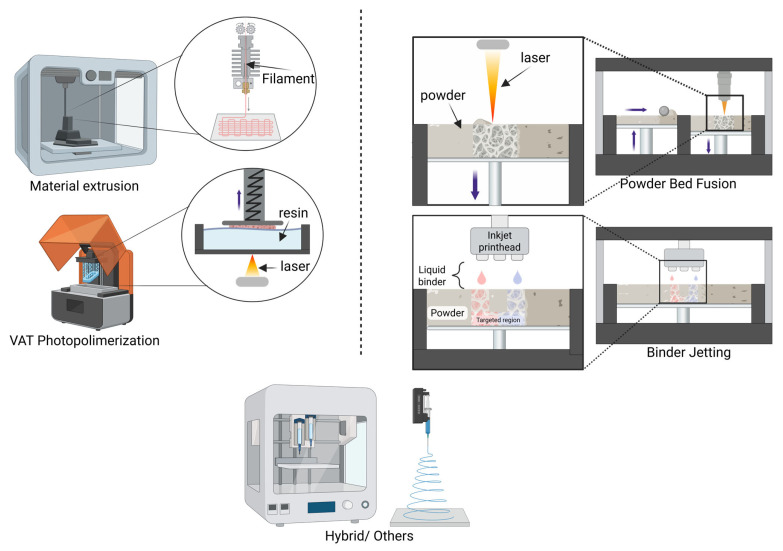
Schematic representation of the four major types of additive manufacturing (AM) technologies commonly used in pharmaceutical applications in bone disease modeling: Material Extrusion (ME), Vat Photopolymerization (VPP), Powder Bed Fusion (PBF), and Binder Jetting (BJ). Each technology is illustrated with its respective solidification and material deposition mechanisms. Also illustrated are hybrid/emerging AM systems, representing combinations of multiple printing modalities and advanced fabrication approaches. Created in BioRender. Timofticiuc, I. (2025) https://BioRender.com/5lvi4qg. Date of last access–5 September 2025.

To address the current challenges in treating bone disease, new drug development approaches have emerged. Unlike conventional manufacturing methods, such as compression molding, granulation, and coating technologies [36,37,38], which lack the flexibility to produce unique dosage forms or release therapeutics in a complex profile, 3D printing offers a wide range of additive manufacturing technologies [39]. The American Society for Testing and Materials (ASTM) categorizes additive manufacturing into seven process types [40], of which we identified four main classes most frequently used in drug delivery systems and drug screening platforms for bone diseases: Material Extrusion (ME), Vat Photopolymerization (VPP), Powder Bed Fusion (PBF), and Binder Jetting (BJ) (Figure 1).

One of the more common processes of AM is Material Extrusion (ME) [41], in which a continuous filamentous material is extruded from a heated nozzle and deposited layer-by-layer to create three-dimensional objects [42]. The most common subtype of ME processing is Fused Filament Fabrication (FFF) [43], also known as Fused Deposition Modelling (FDM), which has been used for the fabrication of non-woven bone scaffolds using various thermoplastic materials (e.g., PCL, PLA, and PLGA) [44,45,46]. While the process parameters influence the mechanical properties in the final structure, [47] layer thickness, filament orientation, and inter-layer adhesion involvement lead to different mechanical properties in these scaffolds, and their final physical properties may differ from the biologically-inspired scaffold material [47]. In addition, extrusion bioprinting is another method of Material Extrusion that uses pneumatic or displacement-driven systems to extrude bioinks that contain living cells and growth factors at physiologically relevant temperatures, producing high-fidelity, multi-material soft and hard tissue scaffolds, including articular and osteochondral constructs [43].

Vat Photopolymerization (VPP) is another additive manufacturing (AM) process that can create spatial objects with enhanced precision and complex geometries. This technique is based on curing a light-sensitive resin layer-by-layer with a UV light source [48,49]. A well-known 3D printing technique from the VPP group is stereolithography (SLA), which was the first 3D printing technology to be commercialized in the late 90s and continues to be improved by advancements in optical components for better fidelity [49,50]. The resins used in VPP usually consist of monomers (that can be methacrylated for enhanced mechanical characteristics), photoinitiators, and additives, which polymerize into a thermally and mechanically stable construct [48,51].

The other technique in this group is Digital Light Processing (DLP), which utilizes a digital projector to cure an entire layer of resin at once, thus enhancing print speed and scalability [50,52,53,54,55,56]. In Powder Bed Fusion (PBF), a laser or electron beam selectively melts and fuses powdered materials, typically metal, but can also include polymers, ceramics, or composites, layer by layer [57,58,59]. The unsintered powder supports the part being manufactured [60,61]. PBF methods are limited to medium or smaller parts and require post-processing, for example, powder removal, sintering, and finishing [58,59,60]. Other PBF methods, such as SLS, SLM, DMLS, and EBM, offer flexibility to the materials and structural complexity [57,58,59,60,61,62,63,64]. Moreover, Binder Jetting (BJ) uses a print head to selectively deposit a liquid binder (with a volume around 5–15%) onto a powdered bed, bonding the materials under mechanical pressure without heat and enabling processing at room temperature [65,66,67]. This involves the use of water-based or solvent-based inks that can be used for sensitive materials like hydrogels and biologics in tissue engineering and drug delivery applications [67]. Moreover, as in PBF technology, unsintered powder acts as an internal support material, meaning that additional supportive structures needed in AM processes are eliminated, enabling complex geometries [68,69,70]. Although BJ is limited in terms of lower resolution (20 to 100 μm) and post-processing requirements and potential porosity, this technology can represent an alternative if complex constructs with enhanced fidelity are desired [65,66,67,69,70,71,72]. The purpose of this review is to analyze all the possibilities of locally delivering drugs or testing new medications or doses through 3D-printed models or platforms made of biocompatible materials. Although an exhaustive search was conducted for this work in this area, no studies were found on humans; instead, only in vitro or in vivo studies (using animal models) were identified. Given that the initial conceptualization included the expectation of finding results from human studies, it is worth noting that research in this field has not yet reached that stage. Thus, the present review is focused on the clinical translation of the methods discovered in vitro and on animal models, but also on how they can represent an alternative to the current drug testing technology, and in some situations, how they can completely eliminate the need for testing on human patients.

## 2. Methods

A literature search was conducted in the following electronic databases: PubMed and Scopus. The search was conducted in July 2025, using specific keywords, Medical Subject Headings (MeSH) terms, Boolean operators (AND”, “OR”, and “NOT” to eliminate literature reviews), and specific search field tags.

The search was composed of three search queries. The first search query was focused on finding any bone disease by using the structure “osteo*”, which resulted in osteosarcoma, osteomyelitis, osteoporosis, etc. Moreover, in the first query, other terms for chronic diseases with can affect the bones were added by using the Boolean operator “OR”: rickets, Paget’s disease, achondroplasia, fibrous dysplasia, cleidocranial dysplasia, spondylitis, Ewing, chondrosarcoma, and myeloma. The second query was focused on 3D printing or 3D bioprinting and other synonyms such as “additive manufacturing”, “printing”, “three-dimensional printing”, or “3D biofabrication”. The third search query was used to restrain the search area for drug delivery platforms or drug screening models (“drug delivery platform” [TiAb] OR “drug” [TiAb] OR “drug delivery system” [TiAb] OR “drug screening” [TiAb] OR “drug delivery” [TiAb] OR “disease modeling” [TiAb]).

The search was not restricted to any timeline. Articles were included only if available in full-text, in English, and if they were original research. Research was excluded based on title and abstract if it did not involve 3D printing or 3D bioprinting, if 3D-printed constructs were used only as surgical guides or medical accessories, or if the implant was not entirely 3D-printed. Any review articles, editorials, case reports, book chapters, meta-analyses, or systematic reviews were excluded.

## 3. 3D-Printed Drug Delivery Systems

### 3.1. Antibiotics

Osteomyelitis is a critical disease with a high risk of amputation. Even in the most usual manifestations, the occurring critical bone defects exceed the body’s ability to self-heal [73]. The development of local delivery systems was encouraged by the risks and variable efficiency of systemic antibiotherapy administration. In the last 20 years, the most used delivery material for antibiotic release has been polymethyl methacrylate (PMMA) [74]. 

**Figure 2:**
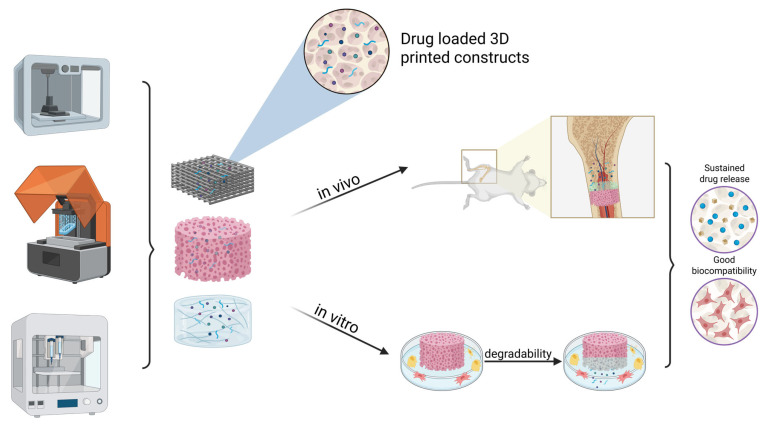
Diagram depicting evaluation pathways of 3D-printed drug-loaded constructs. Each construct produced by a different additive manufacturing technology is examined in an in vitro/in vivo setting for properties such as biocompatibility, pharmacokinetics, and therapeutic efficacy. These constructs can allow a sustained drug release upon implantation and exhibit good compatibility with bone tissue for localized and effective treatment of diseased bone. Created in BioRender. Timofticiuc, I. (2025). https://BioRender.com/pacx9kq. Date of last access–5 September 2025.

However, the lack of control on the PMMA release behavior resulted in many aggravated cases of chronic osteomyelitis or even sepsis [75,76]. To overcome this challenge, 3D printing technologies were used to create a customizable, specific, and controllable drug delivery system for a variety of antibiotics (Figure 2). Advantages and challenges of unique scaffolds are discussed below, offering a broad view of the current research in patient-specific 3D printing-based antibiotherapy.

#### 3.1.1. Vancomycin

Vancomycin is a frequently used antibiotic in the field of orthopedics, particularly for severe implant-related infections caused by resistant gram-positive bacteria such as methicillin-resistant *Staphylococcus aureus* (MRSA) [77]. This antibiotic binds to a highly specific peptide terminal (D-Ala-D-Ala dipeptide) of the peptidoglycan precursors. By inhibiting this terminal, vancomycin stops the crosslinking of peptidoglycans and ultimately inhibits the bacterial wall synthesis [78]. Vancomycin dosing is complex, so therapeutic drug monitoring is essential, especially in patients who are in critical condition [79]. A good control of the release can minimize the risk of toxicity or adverse reactions. Furthermore, given the extensive pharmacokinetics of vancomycin in adult and pediatric populations, an individualized and highly specific dosing protocol is considered the gold standard [80,81].

If a severe infection or a local trauma occurs, the vascularization of the affected area is deficient [82,83]. In these cases, systemic administration of vancomycin would be highly inefficient, and achieving high concentrations of the antibiotic in the affected zone is improbable. Therefore, it is necessary to develop more efficient methods for local and controlled antibiotic administration systems. Various non-3D-printed scaffolds made of hydrogels or polymers, such as gelatin or tricalcium phosphates, were tested as vancomycin delivery systems [84]. However, for these standard scaffolds, the degradability rates, pore sizes, and other mechanical aspects (viscosity, strength, and elasticity modulus) are not controllable. Because of this, the drug release rate can be inconstant; a low degradability rate would directly affect the release, and a lack of control on the mechanical aspects could result in collapsed structures inside the scaffold.

In an in vitro study, a 3D bioprinting gelatin methacrylated (GelMA) scaffold for vancomycin delivery was tested. To solve the high degradability rate of the hydrogel, which would have rapidly released the entire quantity of the drug, zinc-based metal-organic (ZIF-8) nanoparticles were added. ZIF-8 nanoparticles provide supplementary crosslinking points during the biomaterials polymerization process, which enhances the overall rigidity [85]. As a result, the mechanical strength increases, and the early degradation is prevented [86]. Furthermore, it was discovered that at an optimal concentration of ZIF-8 of approximately 3 mg/mL, the nanoparticles exhibit high cytocompatibility and promote osteogenesis by stimulating RUNX2 and OPN, key regulators for osteogenic stem cell differentiation [86]. Therefore, a hydrogel-based scaffold with osteogenic properties and enhanced mechanical characteristics was printed, resulting in a tissue-engineered construct for bone regeneration that is optimal for vancomycin release and simultaneously exhibits antimicrobial activity. While vancomycin-loaded GelMA/ZIF-8 scaffold presents enhanced osteogenesis and antimicrobial properties, in vivo activity and long-term cytotoxicity remain to be studied.

Vancomycin-loaded hydrogels were also tested in vivo, in white mice models, for treating osteomyelitis. The main challenge when inserting a scaffold inside animal models is that the control of pH is lost, as opposed to in vitro testing. For this, the hydrogel (chitosan) was cross-linked with glutaraldehyde, thus ensuring a pH and temperature-responsive scaffold [87]. By the dual incorporation of the chitosan (chitosan loaded with vancomycin and chitosan-laden microspheres), a controlled release of the drug is ensured while also promoting osteogenesis. Moreover, by including the microspheres, a “shield” is created that protects the osteoblast from bacterial toxins, while vancomycin provides antimicrobial activity [87]. However, glutaraldehyde is a crosslinking agent that is considered toxic and carcinogenic. Therefore, an adaptation or substitute that ensures the cross-linking of the hydrogel needs to be identified, especially if the scaffolds are meant to be used in human patients [88,89,90]. One good example of an alternative agent is genipin, a plant-derived substance. It was shown that genipin, used as a cross-linking agent for chitosan, can enhance mechanical integrity and cytocompatibility, thus making it a better candidate for a chitosan-based drug delivery system for clinical use [91].

Chitosan can also be mixed with polymers for enhanced bioactive properties and osteogenesis. For example, in vitro studies revealed that constructs made of PMMA/PCL (polycaprolactone) and chitosan presented a plateau release of vancomycin after 24 h, while also stimulating human bone marrow stem cells [92]. PMMA and PCL are used for their capacity to create optimal pore sizes for better stimulation of the surrounding cells, and the chitosan, as mentioned earlier, is a perfect carrier for antibiotics, especially vancomycin. The rugosity of the polymer scaffold managed to offer a good adhesion to the chitosan coating, which offered a controlled release without premature degradation [92]. Good release of vancomycin and enhanced antimicrobial activity, in vitro, were also obtained with the following 3D-printed composites: PCL and silica [93], methylcellulose and hydroxyapatite (HA) [94], and PLA (polylactic acid)/HA and chitosan [95].

Metals can also be used for a drug delivery system for vancomycin. In an in vitro study, Titanium-Tantalum-Niobium-Zirconium (TTNZ), a metal alloy, was mixed with hydrogels (TTNZ-Chitosan-Hyaluronic Acid) and loaded with the antibiotic [96]. By testing different concentrations of vancomycin, the optimal concentration for antimicrobial activity and cell adhesion was 5% wt; furthermore, it was proven that the hydrogel loaded with vancomycin, when loaded in the porous structure of TTNZ, led to a slower but sustained release rate [96]. This represents a promising result from a clinical perspective, especially if a long prophylactic treatment is desired, rather than a critical infection treatment, where a high burst of drug release with a shorter period of plateau would be required. Metal-based drug delivery systems for vancomycin were also tested in vivo on New Zealand white rabbit models, where another titanium alloy was used (Ti6Al4V) [97]. Besides the already proven antimicrobial efficiency of these locally administering systems, the porous structure of this metal also promoted bone formation in the femoral defect in rabbits [97]. A strong rationale for choosing metal-based delivery systems, rather than hydrogels or polymers, is due to the ability to withstand very high loads [98]. If a custom-made full-prosthesis that also acts as an antimicrobial system is required, titanium can be a good choice.

A variety of 3D-printed patient-specific scaffolds were designed, with all existing categories of materials from the medical 3D printing industry (biopolymers, hydrogels, ceramics, and metal). All materials presented local efficiency and long drug release periods; some also acted as tissue-engineered scaffolds, where new bone formation was promoted. It is therefore the duty of the medical teams and future research to select optimal and specific mixes for the medical needs and requirements of each patient. The major disadvantages of systemic administration disappear when utilizing these solutions; therefore, an improvement in the patient’s life and a decrease in the risk associated with antibiotic resistance or the occurrence of systemic adverse reactions may be expected.

#### 3.1.2. Ciprofloxacin (CFX)

Ciprofloxacin is a second-generation antibiotic of the fluoroquinolone group (1-cyclopropyl-6-fluoro-4-oxo-7-(piperazin-1-yl)-1,4-dihydroquinoline-3-carboxylic acid) that presents a broad antimicrobial effect, and is used in severe infections of various regions such as the respiratory tract, skin, and bone [99]. The main action mechanism against bacteria is via the inhibition of DNA gyrase, but unlike other antibiotics from the same class, it is more efficient against gram-negative bacteria, such as *Pseudomonas aeruginosa*, a known candidate found in foreign body implants, frequently used in orthopedic surgeries [100,101].

Many in vitro studies suggest that CFX is highly effective in treating osteomyelitis [102]. Moreover, many derivatives of CFX have shown great results in achieving anti-tumoral or anti-oxidation properties, which support the antimicrobial activity and healing [99,103]. Despite its great therapeutic advantages, oral administration can be accompanied by adverse effects with a great impact on post-orthopedic interventions recovery, such as tendinopathy, neuropathy, altered muscular metabolism, or even tendon rupture, along with increased toxicity for some patients [104,105]. As such, a local form of delivery would facilitate the partial bypass of the side effects, and an optimal solution to these challenges stands in personalized, 3D-printed drug delivery systems.

In an in vitro study, a 3D-printed thermopolymer scaffold was designed and loaded with CFX to test its effects in treating osteomyelitis [106]. To ensure a good delivery rate, the thermopolymer mix (biocompatible poly(ethylene oxide terephtalate)/poly(butylene terephtalate)-PEOT/PBT) was chosen accordingly, for its capability of ensuring a mechanically stable interconnected pore network, with a high percentage of pores, a key characteristic for controlled degradation and release of the drug [106]. When tested on human mesenchymal stromal cells, there was no relevant cytotoxicity present, and the release of CFX was sustained for one month after a burst phase in the first 24 h [106]. A stable and controllable delivery rate was, therefore, obtained [106]. Moreover, the polymers included in the scaffold enhanced the regenerative capabilities of the bone. If translated for in vivo animal/human tests, the scaffold might potentially heal large bone defects while ensuring a stable degradation rate for optimal drug release, thus combining targeted antimicrobial activity with bone regeneration.

PEOT/PBT is an elastomeric thermoplastic mix with important properties, such as high elasticity, viscosity, and wettability [107]. The crystal structures incorporated in its structure grant it mechanical integrity and stability, which, alongside the good degradability in an aqueous environment, make it a strong candidate for use in drug delivery [107]. However, many aspects need to be strictly controlled in order to achieve the desired construct, especially if the translation into clinical practice is desired. Moreover, thermoplastics are known mainly for their physical, rather than their biological and physiological properties [107]. In this context, other materials may be more appropriate for in vivo animal/human implementation. One example is silk fibroin hydrogel, a functional, bioactive material that can be transformed into various 3D bioprintable inks with increased strength, self-healing, adhesive, and conductive properties for sustained drug release or any combination of properties that is desired, depending on the mechanical or bioactive needs [108].

In this spirit, a team of researchers tested a 3D-printed silk fibroin hydrogel loaded with CFX-laden silica microparticles, in vitro, on a mouse pre-osteoblast cell line (MC3T3-E1) [109]. Alongside the antimicrobial effects, the integration of CFX in microparticles improved the control of drug release, lowering the burst phase time to 4 h compared to 24 h when using thermoplastic scaffolds [109]. Moreover, a very high cumulative release rate of approximately 50% was obtained after 72 h, proving that incorporating the CFX in particles is also effectively limiting scaffold-associated infections through its high release rate [109]. The innovative feature of this study is the synergistic combination of bone healing and bone inductive properties of the hydrogel scaffold with CFX’s antimicrobial activity. The increased surface rugosity and enhanced osteoinductive and osteoconductive properties of silk fibroin can play a key role when integrated in large bone defects caused by bone infections in human patients [109].

CFX can also be integrated into 3D-printed drug delivery systems in combination with other drugs, if more pharmacological effects are needed. Mixing it with other antibiotics for effect-compounding, such as gentamicin, or with anti-inflammatory drugs, such as dexamethasone, are just some examples [106,110]. Many other materials can be adapted for testing different delivery rates or other dynamics of the CFX. One good example is poly-caprolactone (PCL), a biocompatible and bioactive polymer with broad applications in 3D-printed scaffolds used in orthopedics, both in vitro and in vivo (animal models–white rats/rabbits) [110,111]. Using these approaches, high-precision, customizable, and locally effective systems can be obtained, allowing medical teams to tailor the antimicrobial treatment to each patient’s needs, aiming for effective recovery and healing, and reduced cytotoxicity and side effects.

#### 3.1.3. Gentamicin

Gentamicin is an aminoglycoside widely used in the treatment of pseudomonal osseous infections and acts by binding to the 30S ribosomal unit of the bacteria, inhibiting protein synthesis [112]. Gentamicin is also frequently used in the treatment of orthopedic implant-related infections [113]. Regarding its safety, literature studies frequently report nephrotoxicity in cases of systemic administration, which imposes the need for an alternate route [114]. A large number of locally controlled delivery systems were clinically used to achieve high concentrations of antibiotics with low systemic absorption. The most intensively studied is the loading of gentamicin in a biopolymer, usually PMMA, that will be molded in a specific shape desired by the medical team for treating complex bone defects [113].

However, some studies conducted on standard medical biopolymers suggest that if the drug remains fixed inside the implant, usually due to a low degradability rate, the antimicrobial effect is minimized, and due to the surface roughness, bacterial colonization is facilitated, thus generating a high risk of sepsis or amputation [115]. Given that, other methods of drug delivery and various types of materials were tested. With the emerging field of 3D printing in medical applications, personalized and highly controlled release systems, such as specific porosities, mechanical properties, and degradability rates, were newly designed or optimized and tested in vitro, which could play an important role in future clinical practice [116,117,118].

Besides the infection cases caused by an insufficient release rate of antibiotics from PMMA-cement, many studies reported localized thermal necrosis due to the high exothermic polymerization when the cement was used in vivo on human patients [119,120,121]. By 3D printing PMMA in specific morphologies, there is no longer a need to manually shape the cements intraoperatively. Thus, the risk of thermal necrosis is eliminated because all the heat-generating reactions have already taken place preoperatively [118]. In an in vitro comparative study, PLA and PMMA scaffolds were designed and loaded with various antibiotics such as gentamicin, nitrofurantoin, and tobramycin [118]. The most effective system was the PMMA scaffold, which allowed for a controlled release rate of gentamicin and tobramycin; however, in the case of nitrofurantoin, only low effectiveness was achieved. The study proved that optimization of PMMA delivery shapes can be achieved using 3D printing with beads, disks, or layered filaments, and various antibiotics can be released without the disadvantages of the standard techniques [118]. Similar to PMMA, PLA scaffolds were loaded with the same antibiotics. While gentamicin release was not as clinically relevant as PMMA, with antimicrobial activity performing optimally only at a concentration 2.5 times higher than PMMA scaffolds, PLA managed to perform in releasing nitrofurantoin [118].

The main cause of the different behavior between the two similar biopolymers could reside in the extrusion temperatures. The range of temperatures for extruding PLA is around 175–275 °C, while for PMMA, it must be lower than 240 °C [122,123]. Therefore, another highly relevant aspect when loading antibiotics into polymers that are later extruded is the melting point of each antibiotic. For example, tobramycin, which has a melting point of around 200 °C, could only be printed optimally with PMMA scaffolds, because PLA requires higher temperatures for increased precision [124]. This can also explain the need for a higher concentration of gentamicin when released from PLA due to thermal inactivation of the antibiotic compared to PMMA. However, compared to PMMA, which already has clinical applications, the use of PLA for drug delivery should be further studied in vivo on animal models to gain a deeper understanding of how physiological microenvironments affect the release behavior.

Gentamicin can be used in combination with other drugs for immunosuppressed cancer patients. Methotrexate, an antifolate drug, is used in the treatment of various cancers, but similar to gentamicin, it can exhibit nephrotoxicity [125]. In severe cases of renal dysfunction, the systemic administration of these two drugs would be imprudent. However, when released controllably from a local scaffold, the risk of systemic absorption is substantially lower. In an in vitro study, the controlled release of gentamicin and methotrexate from a PLA-based 3D-printed scaffold was researched. For optimal release of the drugs, different printing temperatures were used compared to previous studies. A lower quality of the scaffold was preferred; thus, lower temperatures were tested, 175 °C for the antibiotic and 160 °C for methotrexate [117]. The 3D-printed scaffolds presented good release that inactivated the in vitro culture of osteosarcoma cells loaded with *E. coli* vitroids [117].

Other studies tested the same combination of drugs and demonstrated that an optimal release for 30 days can also be achieved if other materials are used, such as a composite PCL-HA [116]. The advantage of this composite is that the ceramic (HA) can be easily dispersed inside the PCL pores, which are usually formed at high temperatures, thus directly influencing the cellular activity and the drug release in a more controlled manner [126]. This type of polymer-based scaffold, which frequently requires high temperatures, is optimal for the release due to the feasibility of pore formation, a key element in the degradation of the scaffold that ensures the drug elution. However, to adapt this type of construct to a temperature-sensitive drug, some adjustments to the mixture need to be further researched. One example in this direction could be the addition of hydrogels to the mixtures; due to their properties of transitioning from liquid to gel states with temperature increase, lower printing temperatures for the entire composite can be achieved [127,128,129,130].

#### 3.1.4. Tetracyclines

Tetracyclines represent an extensive class of antibiotics, with broad antimicrobial activity on both Gram-positive and Gram-negative bacteria [131]. Tetracycline, a first-generation representative, presents limitations regarding pharmacological bioavailability and pharmacokinetics [131]. However, the advantage of using tetracycline in orthopedic applications lies in its capacity to generate an osteoconductive interface. Therefore, it minimizes the bacterial colonization on the implant surface while also promoting osseointegration [132]. Moreover, some studies suggest that tetracycline can inhibit some matrix metalloproteinases, key enzymes for soft tissue regeneration, and can serve as a healing agent for tendinitis or articular defects [133]. Several customized 3D-printed drug delivery systems were created for delivering tetracycline.

A team of researchers developed a new material mix for a fused-deposition material (FDM) 3D printer [134]. By mixing thermoplastic polyurethane and polyvinyl alcohol, they created nanoporous filaments that presented good degradability and biocompatibility [134]. Using these novel filaments for the construction of the delivery scaffold, a drug system for tetracycline capable of both drug release and absorption was created. The limitation of this study is the short period of 3 days in which the release was tested; however, it was shown that the scaffold did not interfere with the cells (human primary fibroblast) life or functionality [134]. Moreover, the drug release remained stable and controllable. The mechanism of pore formation is based on the capacity of the PVA to dissolve in water [135,136,137]; thus, a sponge-like structure is created. The pore formation is not as optimal as in other materials, such as PLA or PCL, which could affect the mechanical integrity [138,139,140,141,142]. On the other hand, creating a polyurethane sponge-like structure offers absorptive properties to the structures, characteristics that are not often possible with other materials [143]. One of the reasons could be due to the elastomeric properties of the thermoplastic polyurethane (TPU); the pores formed by the elution of PVA expand when hydrated in a liquid environment, promoting absorption [144]. This creates the advantage of absorbing any drug like a sponge, without preloading the drug into the materials before printing. Another advantage is the use of FDM for this system, TPU and PVA being considered easy-printable materials [145,146,147]. Even if the study was performed in vitro, the translation to in vivo animal/human testing is feasible given the technology used, the material availability, and the rare characteristics of drug-loading through sponge-like absorption.

Even though first-generation tetracycline presents many advantages and applications, frequent bacterial resistance mechanisms develop with this drug, such as drug efflux pumps [148]. Therefore, efforts were made to develop other generations of tetracyclines. Doxycycline and minocycline are two relevant representatives of the second-generation tetracyclines, and they present enhanced tissue penetration due to the increased lipophilicity, as well as more feasible dosing protocols [149]. Composite materials were created for enhanced properties and tested in vivo on white rabbits with tibial defects. In this case, the loaded drug was doxycycline, and hydrogel was added to the mix for optimal release rate [150]. Moreover, to accomplish a bone defect repair, ceramics (hydroxyapatite) were added, which are known for their great osteoinductive and osteoconductive properties [111]. Photopolymers (PCLs) were also added for optimal pore formation [150]. The scaffold was tested in a wet state, and a higher release rate was observed compared with previous studies; more precisely, the release was controlled for 28 days in vivo, and after only 15 days, a 100% cumulative release was obtained [150]. As mentioned earlier, second-generation tetracyclines are more liposoluble, so this could stand as an argument for a higher release in the wet-state, purely based on the molecular properties and interactions between two immiscible media.

Improving cell adhesion can increase antimicrobial activity and cell proliferation for rapid healing [151,152]. This can be achieved by adding collagen to the composite mixture and was already demonstrated by a group of researchers who tested a minocycline 3D-printed drug delivery system in vitro on human bone marrow stromal cells [153]. The collagen addition did not affect the minocycline release dynamics. Tetracyclines not only exhibit antimicrobial activity like other antibiotics presented in this section but can also promote bone formation and healing. Although it frequently generates resistance mechanisms in bacteria, this could represent an interesting alternative, mainly due to its unique release mechanism. Translation in clinical use remains to be further studied.

#### 3.1.5. Other Antibiotics

Other strategies in designing release systems were also studied. Some cases of chronic osteomyelitis require combined therapy strategies [151,154]. Systems able to sustain a combined and synergic release were created. It was observed that the morphology and structure of the scaffolds play an important role in the tandem drug release capabilities. Therefore, a four-circle concentric system was designed to deliver levofloxacin in combination with tobramycin, and thus the plateau period of release was prolonged to 60 days compared to previous studies where a porous scaffold maintained the release for 30 days [155]. The advantage of using 3D printing for these systems is the possibility to adapt the position, porosity, dimensions, and other mechanical properties for each circle [155]. In conclusion, a complex system is created in which each component can be loaded with a different drug, and control over each release mechanism is established, so that the synergistic effect is optimal.

3D-printed local systems were also designed for patients with osteoarticular tuberculosis. In this case, given that the treatment to kill *Mycobacterium tuberculosis* is a long-term therapy, local administration is essential to avoid systemic side effects [156]. Considering the potential of tissue-engineered scaffolds for the local administration of rifampicin, tricalcium phosphate was added alongside the drug-loaded microspheres to offer absorbent properties to the scaffold, while also promoting the healing of bone defects by enhancing the activity of osteogenic genes ALP, OCN, and BSP [157].

In the case of semi-synthetic antibiotics, such as roxithromycin, the adaptation for 3D printing is challenging. The main factor is represented by the solubility properties of the drug and also of the scaffold. In a study where PCL, a substance with low water absorption and hydrophobicity, was used for roxithromycin release, PEG was added to increase the hydrophilicity for a more sustained release and to enhance the bioactive properties [158]. Even though a constant release was achieved, a drug burst was observed in the first hours in vitro. In the cases where a more sustained release is desired from the first moments of activity, adding hydrogels in a multilayered system can represent the solution to limit the initial burst phase [159]. As long as all physical properties are taken into consideration (viscosity, pore sizes, elasticity, solubility, wettability, etc.), adaptations for good printability to various commercially available antibiotics can be achieved. Numerous “background” mechanisms are of great importance when designing a drug delivery system, the most relevant being cross-linkings, pore formation, degradability, and cytocompatibility, among many others.

### 3.2. Anticancer

Osteosarcoma mainly affects young people and is characterized by rapid invasion and metastasis [160]. The anticancer drug branch is constantly improving its therapy strategies, but the main challenge remains achieving a constant and sufficient drug release that can optimally kill tumor cells [161]. Similar to antibiotics, 3D-printed drug delivery systems can lead to high efficiency in treating osteosarcoma. Moreover, the base principles of 3D printing systems loaded with anticancer drugs do not differ from antibiotic-laden platforms in regard to the 3D printing technologies, materials, release mechanism, or other technical aspects. Given that, only relevant anticancer drugs will be discussed in this section, and the major challenges for each specific drug will be highlighted, pointing out the differences from other drugs in the adaptation for 3D printability.

#### 3.2.1. Cisplatin

Cisplatin is the first choice as an anti-osteosarcoma drug and was tested in vivo on white mouse models [162,163]. Titanium alloy was used to create the load-bearing structure, and through electron beam melting 3D printing technology, a highly porous scaffold was produced [162]. However, highly porous titanium scaffolds are not characterized by absorption or release properties [164]. Therefore, inside the pores, a hydrogel structure (PLGA-PEG-PLGA) was created and loaded with cisplatin. Consequently, a dual system was created, as the hydrogel composite is capable of optimal drug release and has bone formation properties, while the titanium evenly distributes the drug-loaded hydrogel, preventing the whole system from collapsing [162]. Compared to antibiotics, the release balance is more challenging in this case; excessive cisplatin will increase the cytotoxicity, and a low concentration could generate resistant osteosarcoma cells [165]. Moreover, the affected zone could simultaneously be infected with resistant bacteria. The combination of drugs can be achieved, but the release is more challenging [166]. In the case of cisplatin, drug screening models for synergistic effects should be designed.

#### 3.2.2. Doxorubicin

Doxorubicin is a general anticancer drug effective on a broad range of malignancies. However, the systemic administration of this drug can cause cardiotoxicity and myelosuppression [167]. Many categories of the previously presented materials were already designed in a 3D-printed drug delivery system for doxorubicin–metals: titanium alloys [168], Fe_3_O_4_ nanoparticles [169]; hydrogels: GelMA [170], cellulose [171]; ceramics: hydroxyapatite [172]. The major challenge when adapting doxorubicin to 3D printers and local delivery is the low hydrophilicity [173]. Due to low solubility, the water particles cannot penetrate the scaffold to ensure the drug elution [174]. This property directly affects the drug kinetics; thus, for good results, encapsulation is frequently needed [175]. Encapsulating the drug in liposomes or other micellar systems can modify its physical properties and transform its crystalline phase into a more amorphous state, which favors interaction with water [176,177].

Another solution stands in adding hydrophilic additives, and examples in this direction are the addition of clay particles or any bioresorbable materials, known for their capacity to increase the wettability of a scaffold [178,179]. Moreover, balancing the solubility can bypass the premature unloading or burst phase [167]. Generally, for a successful release, the solubility of the released item needs to be higher than that in the media, and when hydrophilicity is significantly increased, burst phases can occur [180]. Even though low solubility is contradictory to the burst phase for doxorubicin, it was shown that an important quantity of doxorubicin gets adsorbed to the surface of some materials, like PLGA, and gets released extensively when in contact with an aqueous medium [181]. The solution to that could be to ensure better homogeneity of the scaffold and to limit the external adsorption of the drug to the 3D-printed scaffold.

#### 3.2.3. Zoledronate

Zoledronate is a drug used mainly for treating osteoporosis, but in the last decade, its anticancer properties drew a lot of attention as an innovative treatment option in osteosarcoma [182]. By inhibiting critical mechanisms for tumor progression, such as angiogenesis and invasion, preclinical studies suggest that it can play an important role in treating bone malignancies, while also repairing the bone defects caused by the cancerous cells [183]. Given its combined effects, it quickly became a great candidate for a tissue-engineered drug delivery system. In an in vitro study, where zoledronate was included in a 3D-printed PLLA scaffold, it was proven that the drug release is pH-responsive [184]. In the acidic media group, the zoledronate release was around 21%, and it sustained for several weeks, compared to the neutral pH group, with a very low rate of release. This property, combined with the powerful ability to inhibit the osteoclast activity, highlights the capacity of zoledronate to act only at the affected area, given that the osteoclast surrounding is acidic [184]. Studies that assessed the zoledronate local drug release in vivo on animal models exist only for loaded coatings [185]. To our knowledge, there are no reported in vivo studies with biocompatible materials laden with zoledronate that perform release by scaffold degradability.

Two main challenges occur when 3D printing with zoledronate. The first concern is the thermal stability of zoledronate, especially for extrusion-based 3D printing technologies that usually use high temperatures [186]. Studies on thermal stability for zoledronate suggest that small alterations appear at around 100–150 °C, and more critical processes for its structure start to occur above 180–190 °C [187]. For most of the materials compatible with extrusion-based 3D printers, the optimal printing temperature is above 200 °C [188]. As a solution for this issue, 3D-bioprinting in combination with different hydrogels with tailored properties, a method already described previously for thermal-sensitive antibiotics, can be used. Moreover, it was observed that due to the high polarity of the drug, it can interact with the polymers and affect its solubility and printability [189], which will directly impair the drug release and scaffold morphology, especially regarding pore sizes and homogeneity. These aspects were observed in in vitro studies or in literature studies on zoledronate and were not yet approached by the researchers in their attempt to adapt zoledronate to 3D printers. Therefore, thermal stability and polarity remain to be studied in further research.

### 3.3. Anti-Inflammatory and Other Drugs

Recent authors have studied synergistic effects of different mixes with antibiotics and anticancer drugs, attempting to directly solve the most serious bone diseases. Among the groups of drugs added as secondary products to better treat the aforementioned diseases, adaptations have also been made to create 3D drug delivery systems for anti-osteoporotic drugs, anti-inflammatory drugs, or even natural compounds with an antioxidant role. A frequently used anti-inflammatory drug is dexamethasone, a synthetic corticoid that also acts as an immunosuppressant and decongestant [190]. Dexamethasone is very sensitive to UV light, which can greatly affect its structure and pharmacological activity. Thus, when UV-based 3D printing technologies are used, higher concentrations of photopolymers need to be used for the scaffold compared to antibiotics or anticancer drugs. Therefore, scaffolds for dexamethasone delivery are usually made of polymers that protect the drug from UV denaturation, such as PTMC (poly (trimethylene carbonate)) [191] or PCL [192]. Other anti-inflammatory drugs that can be incorporated in 3D-printable systems made of hydrogels or polymers are ibuprofen and aspirin [193,194,195].

In some of the antimicrobial or anticancerous 3D-printed drug delivery systems, an antiosteoporotic drug or bone growth factor can be added to regenerate the affected bone area while also resolving the main cause of the defect. The approach should consider whether the drug has low solubility, therefore requiring encapsulation, or if the drug is thermosensitive, so then hydrogel can be added, or if the scaffold has low mechanical strength, so then metals or ZIF-8 can be used. Using this method, alendronate, bone-morphogenetic protein-2, and D3 vitamin were also included in systems for improved bioactive effects and healing in bone defects caused by infections, cancer, or osteoporosis [196,197,198,199,200,201]. Most of them were tested in vitro, and they did not show any different results when compared with the 3D-printed systems that were loaded with only one substance, either an antibiotic or an anticancer drug.

The same challenges regarding printability can appear when natural compounds are integrated into 3D-printable mixes. However, even though adapting them is highly challenging due to their complex bioactive properties, some studies presented successful results, but further research is required. As examples, we mention cucurbitacin, a natural compound that promotes angiogenesis, bone formation, and anti-cancer effects (integration into PLGA/TCP was successful with a controlled release rate of 1.70% each day) [202]. Curcumin, with in vivo osseointegration and in vitro chemoprevention, was successfully integrated in a TCP scaffold alongside allicin, another natural compound with antimicrobial activity, obtaining a controlled release for 30 days at a cumulative rate of 22% [203]. Using TCP 3D-printed scaffolds, other antioxidant natural compounds such as gingerol and allicin were integrated and locally released [204,205].

The many existing 3D printing technologies offer broad possibilities for material adaptations in order to sustain different releases of drugs, proteins, growth factors, or other substances. As long as good control over the solubility, printing temperatures, degradability rates, mechanical characteristics, and chemical properties can be achieved, successful studies could be done in future research.

## 4. 3D-Printed Drug Screening Models

In the pharmaceutical field, there is a constant need to produce new drugs with more targeted effects and fewer adverse reactions. Moreover, drugs already in use must constantly be improved, especially regarding their pharmacokinetics and pharmacodynamics [206]. The pharmaceutical industry classically uses two methods to verify the properties of drugs, as well as to verify their entire behavior when in contact with living elements [207]. Animal models and 2D cell cultures have been the central approach for drug testing for the majority of drugs [208,209]. Classic 2D cultures consist of a layer of cells attached to a hard surface, onto which different concentrations of various drugs can be administered. Through their interaction with cells, the degree of toxicity can be observed, and the method is reproducible and relatively easy to perform as a first-stage research in drug testing [210,211]. However, cell–cell and cell–microenvironment interactions under the drug influence are impossible to test with these 2D models. Moreover, there is a lack of architectural mimicry of tissues; thus, results regarding the drug activity can be mistaken [212,213].

Comparatively, animal models can provide the complexity of tissue activity and interactions and can play a key role in preclinical drug testing. However, in many cases, there are differences in the cellular metabolism in humans compared to animal models [214]. This difference can lead to mistaken results regarding physiological activity and cytotoxicity of a given drug. Moreover, animal models are not a feasible choice at present, given the costs, the long period necessary for experiments, and ethical considerations [207]. 3D printing technologies have emerged even in the pharmacological drug testing area as a solution to the drawbacks of classical methods. Multicellular, multilayered, and patient-specific models can be created using 3D printing [215]. Moreover, through 3D bioprinting, cells can also be printed homogeneously and in a controlled manner [216].

**Figure 3:**
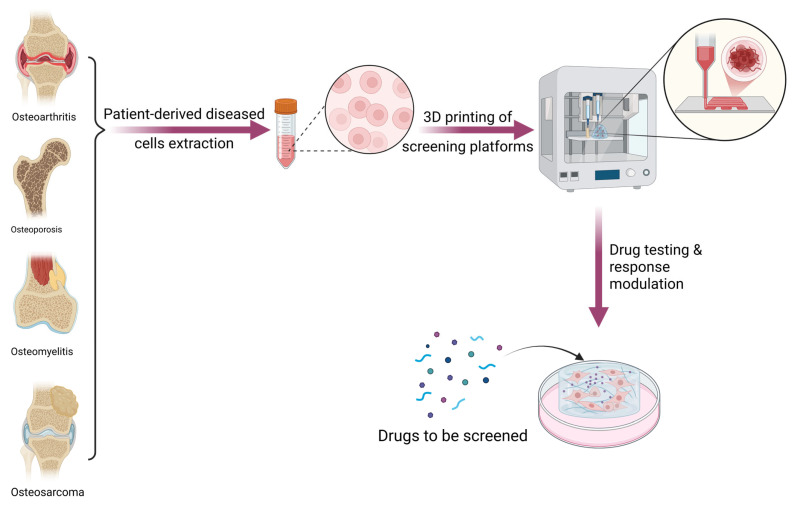
Illustration of drug screening workflow for bone-related diseases such as osteoarthritis, osteoporosis, osteomyelitis, and osteosarcoma. Diseased cells are extracted from patients and 3D bioprinted alongside biocompatible materials, resulting in platforms closely mimicking the bone microenvironment. These customized models are then used to test and modulate responses to various therapeutic candidates, enabling high-throughput, physiologically relevant, and patient-specific drug evaluation. Created in BioRender. Timofticiuc, I. (2025) https://BioRender.com/bwgn10n. Date of last access–5 September 2025.

3D printing drug screening models aims to replace the need for conventional laboratory drug tests or animal models (Figure 3). Moreover, by generating complex 3D architectures, a more faithful model of the human physiological morphologies can be achieved. In particular, 3D-printed models and/or other organoid models could be suitable for mechanistic drug screening, cell–matrix interaction studies, and human cell-based pharmacodynamic testing, where human cellular responses and tissue-specific microenvironments are critical. The major advantage is that the 3D model closely mimics animal or human physiology, is customizable, and can accurately mimic a tissue. Furthermore, it has the advantage of being reproducible and easy to modify. The same model can be printed at any time to test multiple drugs under the same conditions. Also, certain parameters can be modified at any time to see how changing them can influence pharmacokinetics. These systems can model disease progression, local drug delivery, and bone remodeling dynamics and have the potential to yield more accurate results than 2D cultures.

However, animal models remain essential for the assessment of systemic pharmacokinetics (PK), biodistribution, and whole-body toxicity, which require the integrated physiology of multiple organs and metabolic systems. Thus, although 3D bioprinted bone models used in osteoporosis, osteoarthritis, and osteosarcoma studies can effectively replace early-stage screening and reduce animal use. Currently, 3D models as complementary preclinical tools can bring major advantages to drug studies and decrease the time required for their testing. In order to become complete replacements for in vivo testing, future studies should attempt to develop interconnected systems that also mimic the complexity and multifactoriality of biological communication between multiple organ systems of the body. In orthopedics, 3D models were printed for three main groups of bone disease: osteoporosis, osteoarthritis, and osteosarcoma.

### 4.1. 3D-Printed Drug Screening Models for Osteoporosis/Bone Defects

The first step to creating a drug testing platform for bone diseases is to mimic perfectly the internal porous structure. For this aspect, extensive research was performed on all categories of materials, and porous bone-like structures were successfully created using ceramics (hydroxyapatite, calcium triphosphate), hydrogels, and especially photopolymers (PLA, PCL) [217]. Another key aspect is achieving mechanical behaviors of the scaffold similar to those of natural bone. Even though materials, like metals and ceramics, are known for their bearing-loading properties, precisely controlling the exact values of different physical parameters is challenging [218,219]. A solution to achieve high control of the mechanical characteristics lies in 3D printing technologies based on the water-in-oil concept. With these, high internal phase emulsions are used as inks.

These inks are characterized by concentrated emulsion systems where the dispersed phase fractions go over 74% of the emulsion volume [220,221]. A team of researchers used this approach and created a PCL-PVA scaffold (PVA was used as dispersed phase) that was loaded with the osteoblast MG63 cell line [222]. The scaffold’s pores were similar to those of natural bone, but the main advantage is that by adjusting the emulsion composition, different mechanical properties can be achieved. While certain emulsion percentages were not tested, this PCL-PVA scaffold can perfectly mimic the human bone and can be adjusted to each patient’s mechanical profile. By adding the osteoblast, the pores were populated, and a human-like bone microenvironment was synthesized for in vitro studies. As reported in this study, various drugs can be tested on this platform, especially in terms of cytotoxicity and how the extracellular matrix can be modified under the activity of a certain drug [222]. Similar results were obtained by 3D bioprinting a bone organ model of graphene oxide composite laden with human mesenchymal stem cells instead of osteoblasts [223].

Interesting results were also obtained using 3D bioprinters for scaffold-free models [224]. Scaffold-free 3D-printed models do not require foundation biomaterials, because the cells rely on their interaction to create their mechanical support [225,226]. Thus, a more native-like model can be achieved. This approach was used by a team of researchers to specifically test drugs for osteogenesis control on differentiated bone marrow-derived mesenchymal stem cells [224]. While the effects on experimental drugs for osteogenesis inhibition (PD98059 and U0126) were studied, the emphasis was on drugs that promote osteogenesis (Icariin and Purmorphamine). It was shown that purmorphamine, a purine derivative that promotes osteogenesis by Hedgehog pathway signaling, can increase bone formation similar to BMP4 (bone morphogenetic protein 4) [224]. Icariin has also been tested and considered an efficient anti-osteoporotic drug, given the in vitro results.

To conclude, there are already good results in creating organ models that mimic the human bone. Due to the enhanced control offered by the 3D printing technologies and the possibilities to add cells in a precise manner, models that accurately mimic the bioactivity and morphologies of the bone can serve as drug screening platforms. Therefore, the classical testing platforms or the use of animal models can safely be replaced as a preclinical phase component before testing on humans.

### 4.2. 3D-Printed Drug Screening Models for Osteoarthritis

Another form of scaffold-free 3D bioprinting is creating spheroids instead of cell sheets, which was attempted for osteoporosis models. Using the same principle, by aspiration-assisted 3D bioprinting techniques, cells can be placed in certain positions to facilitate their interaction to create self-assembled spheroids [225]. Differently, they create the extracellular matrix on the surface, and the interactions between the tested drug and the scaffold are closer in results to how they would act on real tissues. As such, the drug will reach each cell in different concentrations, depending on its position compared to the interface of interaction [227,228]. Using this concept, a team of researchers created spheroids from amniotic fluid-derived mesenchymal stromal cells, and a chondrogenic-like tissue was formed [229]. Although not tested in vitro or in vivo, it could act as a drug screening platform for osteoarthritis drug in vitro testing for local activity, like sclerostin, cyclopamine, osteoprotegerin, etc. [230].

Advanced systems that can behave as native tissue environments were also designed and created. One example in this direction is bioreactors, which are platforms that allow for various models to be cultivated, such as spheroids, organ-on-chip, and other organ models. They can enable dynamic systems by providing control over the fluids added to it, or the nutrient and metabolic exchanges [231]. Moreover, mechanical stress can be tested by changing the bioreactor environment’s mechanical characteristics [232]. These systems can also be 3D-printed, and an osteochondral bioreactor with fluid control was designed and printed using photopolymer resins by stereolithography [232]. Given that the fidelity of the bioreactors is higher compared to static models, they can also stand as possible drug screening systems for osteoarthritis.

### 4.3. Others

Osteosarcoma models were also 3D-printed, in order to test various anticancer drugs, already mentioned in the drug delivery systems section, such as cisplatin and doxorubicin [170,233,234,235,236]. However, given that 3D-printed delivery systems already exist in this case, the screening platforms can be helpful in testing synergistic effects or if some changes are made to the pharmacological properties of the drug. As base principles, the bone model is created in the same manner, with the only difference being the cell lines that are added. According to the previously cited studies, good results were obtained for MG-63, human osteosarcoma U-2 OS and its cisplatin-resistant variant, and HeLa-human bone osteosarcoma epithelial cell line. Using the bone organ models, any drug can be tested, and it is up to the researchers to select the drugs, cell lines, and materials to use for optimal results. To conclude, 3D-printed drug screening models can play a role as an intermediate test platform between in vivo studies on humans and in vitro studies.

In the next table (Table 1), we have integrated the research conducted on 3D printing, aiming to create either drug delivery systems or drug screening platforms. For each system, the technology, the materials and their properties, the types of drugs and their bioactive properties are specified.

## 5. Future Perspectives and Discussion

The 3D-printable drug delivery and drug screening systems with effects on the main orthopedic diseases, such as osteoporosis, osteoarthritis, and osteosarcoma, were presented here. There is a great need for new and therapeutically optimized treatments for a wide range of diseases with secondary pathological effects on the bone. Among these, the most relevant categories are metabolic diseases, which can alter the bone remodeling metabolism, the mineral metabolism, and the intracellular homeostasis; endocrine diseases, such as parathyroid disorders with effects on bone mineral metabolism or even renal diseases, are also of importance [267,268,269]. Given the complexity of the pathophysiological mechanisms underlying the manifestations of these diseases, there is a need for 3D models designated for local administration and screening for drug optimization, without directly affecting humans or laboratory animals through classical methods. An overview of the available technology studies is provided in Table 2.

Gaucher disease is a genetic disorder characterized by a deficiency of glucocerebrosidase, which leads to the accumulation of glucocerebrosides at the intracellular level, in lysosomes of multiple organs such as the spleen, liver, and bone marrow [270,271]. Bone damage is one of the major complications and causes of death, since bone remodeling and vascularization are totally compromised [272,273]. These effects lead to the appearance of chronic inflammation with unbearable pain and multiple pathological effects, ranging from osteopenia to osteonecrosis [274]. The current treatment stands in the restoration of the missing enzyme to reduce its accumulation in the lysosomes [275]. Pain relief and slowing of bone damage were reported with the combined treatment of enzyme replacement therapy and substrate reduction therapy [276]. However, local therapies with drugs for bone repair could present a potentiating element in the treatment of Gaucher disease.

A team of researchers managed to build a bone organ model laden with Gaucher cells. By aspiration-assisted 3D bioprinting (technology already presented in the drug screening section), a free-form spheroidal model was designed [277]. To mimic the affected bone, the spheroids were produced by the self-assembling co-culture of human bone marrow-derived mesenchymal stem cells and peripheral blood mononuclear cells from patients with Gaucher, which were differentiated into bone cell lineages [277]. While no drugs were specifically tested on this model, it could easily act as a drug screening platform where bone-healing medication or Gaucher drugs can be tested, such as monoclonal antibodies or Sclerostin [278,279]. Similar results could be obtained following the same methods for other metabolic diseases.

3D-printed systems can also be created to test potentially cytotoxic drugs. Microfluidic chips and transparent systems can be built using polymers such as PLA and PMMA [280,281]. The microfluidic chips have the potential to create randomly curved channels in which drugs can be loaded and “travel” in contact with the channel walls, where human cells are present [242]. The use of transparent materials allows testing the drug activity through optical methods, such as optical pharmacokinetics systems [282]. This type of model was created with relatively low costs using an FDM printer, where staurosporine, an apoptosis-inducing drug, was tested for its cytotoxic effects against human mesenchymal stem cells [242].

Theoretically, the potential of these highly controllable and customizable systems is limitless. As long as full control over the mechanical and bioactive parts is maintained, spatial models that perfectly mimic all existing tissues and organs can be created. Moreover, by using stem cells or different cell precursors or progenitors, the pathophysiological mechanisms produced by the affected cells can also be mimicked. As long as the properties of the drugs are known in terms of thermal and mechanical sensitivity, any drug can be adapted to 3D printing. By integrating these two concepts, patient-specific disease models can be created to facilitate the identification of customizable treatments. However, some challenges and limitations remain. In vivo studies and clinical translation are insufficiently addressed. Ethical considerations, methodological procedures, manufacturing protocols, legal regulation, and long-term studies are yet to be studied. In contrast, devices that are 3D-printed must be in accordance with applicable quality systems and regulations on medical devices. The U.S. Food and Drug Administration (FDA) states that if products are made through additive manufacturing, this does not change the regulatory pathway and should not fall under a special protocol [283]. All resulting products must comply with applicable Chemistry, Manufacturing, and Control (CMC) standards [283].

Rather, the FDA’s guidance on additive manufacturing emphasizes technical considerations, material quality, print validation and post-processing quality, physical and mechanical characteristics, and biological considerations (e.g., cleaning, sterility, and biocompatibility) [283,284]. In our view, regarding biocompatible materials, each manufacturer is required to provide the necessary documents to prove the safety of their product, and regarding the safety of production, each institution dealing with the production of such 3D-printable devices must think about and submit for approval a workflow with the possibility of verification by an audit or other third parties.

It is important to note that patient-adapted devices require regulatory authorities to review the design process and adaptation processes for use on the patient, not necessarily the individual device. In our opinion, the regulatory paths to be adopted could be similar to those adopted for the approval of the first pharmacoprinted drug by the FDA in August 2015, SPRITAM^®^, that is, complying with the following indications: those from the Chemistry, Manufacturing, and Control (CMC), as well as the 21 CFR 200s and 300s regulations [283,285,286].

In a 2025 article on clinical trials for 3D-printed drugs, it is mentioned that the FDA in the USA and the MHRA (Medicines and Healthcare products Regulatory Agency) in the UK are developing quality assurance and validation strategies for 3D manufacturing [287]. In the European Union, the Quality Innovation Group (QIG) (2025–2027) has been established to prepare a document aimed at integrating additive manufacturing and decentralized production into existing regulations and to encourage early dialogue between innovators and regulators [288]. The QIG focuses on standards (ISO/ASTM 52900 series) and interdisciplinary regulatory committees. These initiatives aim to address current translational barriers, such as reproducibility, lack of validated reference materials, and lack of clear quality control protocols [289]. Major challenges persist in terms of reproducibility, in contrast with 2D models or animal models.

Furthermore, drug delivery systems must be adapted in order to act as implants. In this regard, they must function biomechanically, and a major disadvantage to their integrity over time is biodegradability. However, the degradability of the prosthesis ensures the release of drugs, a key element for such a system. Given this contrast, it is possible that the total replacement of implants with drug-releasing implants is not an optimal solution. However, combined techniques could be advantageous. Integrating the constructions necessary for resistance over time and for biomechanical efficiency with drug delivery systems could be an option. Another option could be the effort of limiting the degradability over time of the entire implant by forcing osteogenesis to restore the integrity of the piece after the total elimination of the drug.

3D printing presents challenges in terms of stability over time and under various conditions that occur during printing, both for the scaffold made of the chosen biocompatible material and for the drugs [290]. To ensure a clinical translation of a 3D-printed product, we must mention that sterilization is mandatory, but this process can deform the models or leave residues. In a comparative study of steam sterilization (temperatures above 105 °C) with hydrogen peroxide or ethylene oxide plasma of 3D-printed models, it was found that steam sterilization at a high temperature significantly deformed models made of thermosensitive materials such as polylactic acid (PLA) [291,292]. Moreover, it was shown that steam sterilization at low temperature (105 °C), plasma sterilization, and gas sterilization preserved the fidelity of the models. However, it is worth mentioning that steam sterilization generally does not work on porous or architecturally complex models, and plasma or gas sterilization is less common in terms of their applicability in healthcare units [291].

The stability of active substances, such as drugs integrated into 3D models, during printing is another key issue. Many drugs degrade at high temperatures or under UV radiation (such as that used in vat-photopolymerization-based printing techniques). A review of 3D printing of thermolabile drugs indicates that semi-solid state extrusion, DLP, Binder Jetting, and SLA allow printing of thermolabile drugs because they avoid high temperatures, although photopolymerization can lead to light-induced degradation [293].

If temperatures frequently exceed 120 °C, this can lead to drug degradation, altered bioactivity, and poor mechanical stability. Therefore, the printing of heat-sensitive proteins, peptides, or antibiotics should be performed using potentially hybrid techniques that combine low temperatures with limited use of radiation to cover a wider range of drugs [294,295]. Researchers should also assess leachables from photoinitiators or unreacted monomers and confirm that sterilization does not alter drug potency or scaffold properties. Stability studies and aseptic printing processes should be included as potential verification methods in future studies [296].

To present in this section the future potential of printing technologies, we must also mention the possibility of 4D printing. By introducing this term, we refer to the potential of 3D models to present properties over time, such as changes in degradation behavior over time and as a function of temperature. In this sense, a review exemplifies the fact that scaffolds with osteogenic differentiation power can be generated that can change their shape and drug release capacities over time, which may have greater efficiency than the classic 3D models that were presented in this review [297]. We should also mention the potential that AI could have both in generating printing algorithms specific to certain materials or drugs, as well as by using AI in the future, potentially to generate 3D designs without human intervention [298].

From an ethical perspective, 3D printing should always be chosen over the unjustified use of animal models, but given that research in this field is still in its early stages, in vivo validation will still be required. There are no methodologies, protocols, or regulations that prove that 3D printing can completely replace the use of animal models. If future tests demonstrate the effectiveness of 3D printing in drug testing and the benefits will be significantly superior to classical methods, 3D printing might represent a new standard. However, to create the most realistic models, viable human cells from generic cultures or from the specific patient must be used directly. This may lead to issues regarding donor ethical considerations and, therefore, to limitations of using human cells on other patients. The lack of long-term studies cannot indicate whether excessive degradability could produce serious effects on the patients in whom these systems are used. Moreover, from a legal point of view, a policy has not yet been created that would assume responsibility for those who produce the 3D model, the hospital in which they would be used, or the manufacturer of the elements used. In the clinic, researchers should implement, as transparently as possible, the use of informed consent to clarify the use of cells for in vitro or in vivo studies (in the case of bioprinting). Anonymization should comply with data protection regulations, such as the GDPR (General Data Protection Regulation), ensuring that patient identities cannot be easily identified [299]. Standards, such as the Guidelines for Human Biobanks and Databases for Genetic Research, ISO 20387 (Biobanking), recommend clear consent materials, coding systems that separate personal identifiers from samples, and governance structures that control access [300]. Researchers should also consider return policies and data sharing agreements. New regulations are emerging that must be approved both for the pharmaceutical side, especially if combinations of drugs are used, but also for the implementation of scaffolds in patients.

## 6. Conclusions

**Figure 4:**
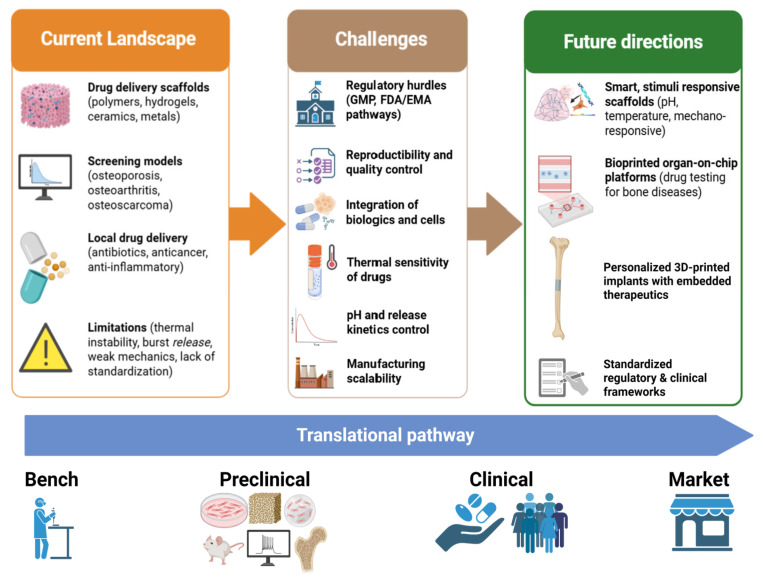
Current landscape, challenges, and future roadmap for 3D-printed bone therapeutics. Created in BioRender. Timofticiuc, I. (2025) https://BioRender.com/7vq75i5. Date of last access–15 October 2025.

3D printing has significant potential in creating personalized drug screening or local delivery systems at the site of the disease. The studies reviewed in this paper present optimistic in vitro and in vivo results for the administration of anticancer, antimicrobial, or bone reconstruction treatments, in a controlled manner. However, translation to patients remains to be further studied, especially from the perspective of reproducibility, regulatory, and also their long-term functionality. The development of new printing techniques and new materials has led drug testing to a possible new standard, from which it can further progress in the direction of personalized treatments in bone therapeutics (Figure 4).

## Figures and Tables

**Table 1:** This table highlights the main results of research conducted on drug delivery and screening systems using 3D printing technologies. The table is categorized by the main technologies, and for each one, the materials from which the scaffold was made, the drug released, and a summary of the properties of the entire scaffold (both drug and material properties) are presented. It is also mentioned whether the study is performed in vitro or in vivo (no in vivo studies on human patients were found).

Broad 3D Printing Technology	Specific 3D Printing Technology	Drugs	Scaffold Material	3D-Printer Commercial Name	Diseases Targeted + Delivery/Screening	Biological and Mechanical Properties	Release Profile	In Vitro	In Vivo	References
Material Extrusion	FDM (Fused Deposition Modeling)	Dipyridamole (DIP)	Thermoplastic polyurethane (TPU)	Flashforge Creator Pro (Flashforge, China)	-Bone defects -Drug delivery system	-Shore hardness–80A -Moderate flexibility -Resistance to deformation -Non-degradable, good biocompatibility -Osseointegration -High cell viability and proliferation at DIP 10% *w*/*w* -High opacity -Compression modulus of 10 MPa	-Slow, sustained over 30 days -10% DIP, the release was ~90% (2888 µg), vs. 5% DIP, where the release was ~3% (58 µg) -No burst	MC3T3-E1 (murine pre-osteoblasts)	Not tested	[237]
		Tetracycline	TPU + PVA porous filaments (PORO-LAY LAY-FOMM 60; LAY-FELT)	Flashforge Creator Pro (Flashforge, China)	-Bone defects -Drug delivery system	-High porosity -Release and absorption properties -High cell viability	Not specified	*Staphylococcus aureus* (cat# 470179-208), Pseudomonas fragi (cat# 470179-090); human ACL fibroblasts (from three patients)	Not tested	[134]
		Nuciferine (NF)	PLA/CS-NF porous scaffold (PLA with chitosan-nuciferine)	Ultimaker 3	-Bone defects -Drug delivery system	-Low molecular weight -75–85% deacetylation -Average pore size: 100–325 µm -Osteoblast differentiation	-Burst day 1, then sustained to 21 days; -68.5–71.8% cumulative by day 21 (dose-dependent 60–100 μM)	Mouse MSCs (C3H10T1/2)	Not tested	[238]
		Prednisolone; Dexamethasone	PLA (Polylactic Acid)	Regemat3D (Spain)	-Bone defects -Drug delivery system	-Osteoinductivity	-Loaded filament scaffolds: <10% in first 2 weeks (sustained) -Post-print loaded: ~50% burst in first 6 h	Murine fibroblasts (CCL-163, ATCC); human bone marrow MSCs (hMSC; ATCC PCS-500-012)	Not tested	[239]
		Minocycline	PLA scaffold coated with type-I collagen + citrate-hydroxyapatite nanoparticles (HA NPs)	BQ Hephestos i3 (Prusa-style)	-Osteomyelitis -Drug delivery system	-Compressive strength: ≈13 MPa -High Young’s modulus values -Low plasticity -Cell adhesion	-Burst within first hour, then gradual 4–24 h	*Staphylococcus aureus*; human bone-marrow-derived stromal cells (hMSCs)	Not tested	[153]
		Gentamicin; Tobramycin; Nitrofurantoin	PMMA bone cement (Orthoset 3) or PLA pellets, silicone-oil coated	MakerBot Replicator (first gen)	-Osteomyelitis -Drug delivery system	Not mentioned	Not specified	*Escherichia coli* and *Staphylococcus aureus*	Not tested	[118]
		Zoledronate	PLA/PCL + HA NPs (indirect: printed + freeze-dried)	ZMorph (indirect printing + freeze-drying)	-Bone defect -Drug delivery system	-Pore diameter ≈ 141.01 ± 48.25 µm -Wall thickness ≈ 27.51 ± 2.94 µm -Porosity ≈ 69–71% -Compressive modulus ≈ 0.6339 ± 0.03995 MPa -Weight loss ≈ 2 ± 2.5% at 35 days; -Slow degradation -Structural stability	Not specified	Not tested	Forty adult males (Wistar rats)	[240]
		No drug (Plasmid DNA encoding microRNA-200c)	PCL scaffold coated with collagen/gelatin sponges	Regemat 3D + Facilan PCL 100	-Bone defect -Drug delivery system	-Pore size 450 µm; -Porosity 55.75%; -Gelatin coating thickness ≈ 17.52 µm; -Basic pH gelatin slowed the release and improved regeneration	-Collagen/acidic gelatin: rapid over 72 h -Basic gelatin: slowed, sustained over 72 h -PCL + polymer coats mimic sponge controls -Burst vs. sustained, depending on polymer	Human preosteoblasts, embryonic palatal mesenchymal (HEPM; ATCC)	12-week-old male Sprague–Dawley rats (N per group = 3–7)	[241]
		Any drug	PLA/PMMA/PC microfluidic chips	Ultimaker 3	-Osteosarcoma model; -Drug-screening platform	Not mentioned	No specific drug tested	SaOS-2 osteoblasts; human MSCs (hMSCs)	Not mentioned	[242]
		Doxorubicin	PLA	Creality Ender 3 Pro	-Osteosarcoma model -Drug-screening platform	-The surface microstructure allowed the optimal development of spheroids and their immobilization	Not specified	Osteosarcoma lines 143B, MG63 (ATCC)	Not tested	[243]
	3D bioprinting	Icariin; Purmorphamine; PD98059; U0126	Scaffold-free-spheroids (bone marrow-derived mesenchymal stem cells (BM-MSC))	Regenova^®^ (Cyfuse)	-Osteoporosis model -Drug-screening platform	-Icariin and Purmorphamine are osteoinductive drugs -Osteogenesis promoters -PD98059; U0126 are osteogenesis inhibitors	Not specified	Bone marrow MSCs at passage 4 (LifeNet Health)	Not tested	[224]
		Vancomycin	Methylcellulose + nanohydroxyapatite (aerogel ink)	Cellink BIO X (BIOX)	-Osteomyelitis -Drug delivery system	-Bone repair and antimicrobial activity -Optimal porosity -Textural stability after 7 months	-Burst first 8 h, then sustained to 72 h	Mouse embryo fibroblasts (NIH/3T3); MC3T3-E1 pre-osteoblasts	Artemia salina eggs; fresh fertilized hens’ eggs; tests in triplicate	[94]
		Pioglitazone (PIO)	nano-attapulgite/PVA/gelatin	Not specified	-Bone defect -Drug delivery system	-High viscosity -Osteogenic differentiation -PIO clear absorbance: 238 nm -Minimal cytotoxicity	-PLGA nanospheres reduced burst; sustained to day 9 (with PNs), total ~32.5% ± 1.5% on day 9 -Without PNs ~41% ± 2.5% at day 5	RAW 264.7 (mouse macrophage); mouse BMSCs (CRL-12424, ATCC); HUVECs	Sprague–Dawley rats, 3-month-old (N = 30)	[244]
		SB216763	PCL	Bioprinter DOME-PCI3D	-Osteoporosis model -Drug-screening platform	-Induces angiogenesis -Osteogenic differentiation (Wnt signaling) -High cell survival rate (91% after 7 days)	Not specified	HUVECs; murine ST2 stromal cell line	Not tested	[245]
		Growth factor (VEGF)	PCL modified with amine groups (CP05)	3D printer (Allcct, China)	-Bone defect -Drug-screening platform	-PCL molecular weight: 50 kDa -Printing temperature: 180 °C -PCL scaffold was optimal for the porous structure of the bone, in which new vessels (due to VEGF) started to form	-Controllable release (qualitative)	Rat BMSCs (rBMSCs)	Male Sprague–Dawley rats (N = 40)	[246]
		Leonurine hydrochloride	Gelatin/alginate + nano-attapulgite	RegenHU 3D Discovery (microvalve)	-Bone defects with vascular impairment -Drug delivery system	-Good biocompatibility -Bone mineralization -Increased blood vessel formation -Tube formation and cellular migration (key elements for angiogenesis)	-Explosive within 72 h, then stabilized -~18% over 17 days (nanostructured scaffold)	BMSCs; HUVECs	Male Wistar rats, 8–10 weeks (N = 50).	[247]
		Dexamethasone (DEX); Amphotericin B; Gentamicin	PCL + nano-HA + alginate/gelatin hydrogel	Two-nozzle pneumatic printer	-Osteomyelitis -Drug delivery system	-PCL-nHA high compressive modulus: 46.37 ± 2.58 MPa -PCL-nHA low compressive strength 4.51 ± 0.47 MPa -Dexamethasone microparticles (MPs-DEX) -spherical: 0.24–5.58 µm -Negative zeta potential of the drug microparticles	-Free MPs-DEX: 55.8% day-1 burst, ~90.4% by day 30. -MPs-DEX entrapped: ~7.0% day-1 then sustained to ~55.8% by day 30	Human endometrial MSCs (hEnMSCs).	Not tested	[248]
		Doxycycline (in DX/HAp/PCL NPs)	Gelatin + PVA + hyaluronic acid; integrated with DX/HAp/PCL NPs	Robota (dual extrusion)	-Osteomyelitis -Drug delivery system	-Osteoconduction; -Bioresorption and immune tolerance; -Pore sizes (90.4 ± 3.9 and 196.6 ± 38.8 µm);	-Wet-state scaffolds: 100% by 15 days -NPs alone: slower (to 24–28 days) -Biphasic 24-day profiles with method-dependent differences	Not tested	New Zealand white rabbits (N = 30)	[150]
		Growth factor (BMP-2)	PCL + mesoporous calcium silicate NPs	BioScaffolder 3.1 (GeSiM)	-Orofacial bone repair -Drug delivery system	-BMP-2 did not affect the mechanical integrity of the PCL scaffold -Porosity: 65% -Pore sizes: 542 ± 13 µm -Good cell viability and adhesion	-Burst day 1 -Sustained, day 14 amount ~4× day 1	Human Wharton’s Jelly MSCs (WJMSCs)	Not tested	[200]
		BMP-2 (in ZIF-8 NPs)	PLGA + MBG + ZIF-8	EnvisionTEC FDM bioprinter	-Bone defects -Drug delivery system	-Compressive strength: ≈2.9 MPa -Increased mechanical strength due to the addition of ZIF-8 -Osteoinductive activity	-Slow; -~40% by 72 h, ~57% by 174 h, continuing thereafter	MC3T3-E1	Sprague–Dawley rats (N = 12).	[199]
		Salvianolic acid B	Alginate/ε-polylysine	BioScaffolder 3.1 (GeSiM)	-Bone defects -Drug delivery system	-Angiogenesis promoter -Suppressed osteoclast activity -+146% bone mass	-~60% cumulative by day 80	C3H10 cells	Female Sprague–Dawley rats, 10 weeks (N = 32)	[249]
		Ibuprofen (IBU)	GelMA + ZIF-8 nanoparticles	Not specified	-Bone defects -Drug delivery system	Not mentioned	Not specified	MC3T3-E1	Not tested	[194]
		Model protein (Cytochrome C)	Sol–gel Ca-doped silica (TEOS/CaCl_2_ inks)	Cellink BIO X (mechanical extrusion)	-Bone regeneration -Drug delivery system	-Calcium increased the pore size (from 1.66 nm to 9.10 nm) -Brittle behavior at 120 N -Adsorption of Cytochrome C of 84% after 5 days -pH-controlled release -By increasing the calcium concentration, larger drug molecules can be released	-pH-dependent -No release at pH 7.5 -Initial burst (24 h) at pH ≤ 5.5, then fast to 72 h -Continued up to 28 d (21 days at pH 5.5).	Rat MSCs (rMSCs)	Not tested	[250]
		Resveratrol (RVS); Strontium ranelate (SrRn)	PCL/hydrogel	Not stated	-Craniomaxillofacial bone defects -Drug delivery system	-Mandibular bone formation after 8 weeks in rats’ mandibular defects -Enhanced angiogenesis -Osteoclast inhibition	SrRn: >70% day 1 burst, then sustained ≥21 days -RVS: <30% over 21 days -Scaffolds are structurally stable over 21 days.	Mouse MSCs; human osteoclasts (from PB monocytes); HUVECs	Sprague–Dawley rats: bone defect only (N = 5), scaffolds only (N = 6), scaffolds + RVS + SrRn (N = 6)	[251]
		5-Fluorouracil	Calcium phosphate cement (CPC)	Bioprinter + Innotere	-Osteosarcoma -Drug delivery system	-Poor solubility of the anticancer drug -Shape fidelity and increased ultimate tensile strength	-Complete release within 2 h for all coated scaffolds	HeLa; HEK293T cell lines	Not tested	[252]
		Parathormone (1-34)(PTH1-34); Simvastatin	GelMA + PLA	Sunp Biomaker Pro (Sunp Biotech)	-Osteoporosis -Drug delivery system	-Mechanical strength similar to natural bone -Stable biodegradation -Porosity rates were around 50% -Compressive modulus of GelMA-PLA: ≈40 MPa -Water absorption of GelMA-PLA: ≈255%	-Simvastatin: 6.29% initial, then gradual to day 19 -PTH1-34 minimal initial, cumulative peak ~day 40 (PLGA microspheres mediated)	MC3T3-E1.	12-week-old female SD rats, sham (N = 3) and OVX (N = 21)	[253]
	Water-based extrusion printing	(general drug-releasing)	PCL HIPE Pickering emulsions (PCL + PVA dispersed phase)	Not specified	-Osteosarcoma model -Drug screening platform	-Good cellular adhesion -Optimal pore morphology that resembles the natural bone	Not specified	MG63 osteoblasts	Not tested	[222]
		SDF-1; Y27632	PU (Polyurethane) dispersion + collagen	Not specified	-Osteoarthritis -Drug delivery system	-Promotion of cartilage proliferation -Cell free scaffold -Stromal cell-derived factor attracted mesenchymal stem cells from the surroundings, which helped in cartilage development	-Y27632 burst at 12 h increases with loading (12–30%) -Then, ~0.10–0.19%/h -Scaffold releases reach effective ~10 µM by 48–62 h	Human bone marrow MSCs (hMSCs)	Adult male New Zealand white rabbits; independent triplicate runs	[254]
	Melt-extrusion	Ciprofloxacin (CFX); Gentamicin	copolymer PEOT/PBT (poly(ethylene oxide terephthalate)/poly(butylene terephthalate))	Not specified	-Osteomyelitis -Drug delivery system	-Interconnected pore network -High porosity -Load-bearing mechanics	-CFX/MgAl: sustained ~1 month -GTM/ZrP: burst <24 h, then slow -Kinetics: Stage I (modified Freundlich), Stage II diffusion-limited; incomplete total release due to polymer diffusion limits	*Staphylococcus epidermidis*; *Pseudomonas aeruginosa*; human MSCs (hMSCs)	Not tested	[106]
	Micro-extrusion	Ciprofloxacin	Methacrylated silk fibroin (SF-MA) + HMSC-MA silica capsules (polymer–silica aerogel)	Micro-extrusion system (name not specified)	-Osteomyelitis -Drug delivery system	-Large bacteriostatic rings -Osteoinductive -Osteogenic	-Burst first 4 h; ~35% by 72 h (most), up to ~50% with higher HMSC content due to high loading (95%) and mesopores	*Staphylococcus aureus* (ATCC 29213); *Escherichia coli* (K-12 DH5α); MC3T3-E1; human MSCs	Not tested	[109]
	Melt Electrowriting (MEW) + FDM (hybrid)	—(BMP-2 in PLGA MicroPs as cargo)	PCL mesh + PLGA microparticles (InductOs BMP-2 formulation)	MEW (outer) + FDM (core)	-Critical-sized bone defects -Drug delivery system	-PLGA allowed the controlled release of rhBMP-2 -Limited ectopic bone growth	-15-day study -Most rhBMP-2 released within 24 h -High-dose MPs ~73% vs. low-dose ~69%; -Amidation did not alter MPs’ kinetics	Human periosteum-derived MSCs (hPMSCs)	Female Sprague–Dawley rats, 8–12 weeks; groups ≥6 animals; randomized double-blind; doses: 0.2–1.2 µg rhBMP-2	[255]
	Melt Electrohydrodynamic Printing	Roxithromycin	PCL/PEG	Custom system	-Osteomyelitis -Drug delivery system	-The addition of PEG increased the hydrophilicity of the scaffold -Increased wettability -Increased cellular proliferation	->70% within 6 h (burst), then slow to 24 h -PEG increased early release (0–15% PEG: 72–88% at 6 h; 76–91% at 24 h)	*Escherichia coli*; *Staphylococcus aureus*; MG63 cells	Not tested	[158]
	Modified/Hybrid/Custom extrusion	Aspirin	PCL	Custom system	-Bone defect -Drug delivery system	Not mentioned	-Sustained release	Human MSCs (hMSCs)	Not tested	[256]
Vat Photopolymerization	SLA (Stereolithography)	Cisplatin	DS-3000 biocompatible resin (DWS)	DWS 028J+, Italy	-Osteosarcoma model -Drug screening model	-Osteosarcoma cells were taken from a patient with residual cancerous cells (HBCx-66) -Pore sizes: 100–500 µm -Young’s modulus: ≈ 2.45 GPa	-Initial burst, then sustained >15 days -Release coupled to PLGA-PEG-PLGA hydrogel degradation within 3D-printed implant	Bone marrow MSCs; breast cancer PDX cells; osteosarcoma (143B, HOS, MG63)	5-week-old female BALB/c nude mice; 5 groups, N = 5/group	[233]
		Bioreactor to test candidate drugs	Somos WaterShed XC11122 resin; agarose porous medium; GelMA hydrogel scaffold	3D Systems Viper si2	-Osteoarthritis model -Drug screening platform	-Fluid velocity inside the bioreactor is tunable to test the drug’s elution -Hydraulic resistance	Not specified	Not tested	Not tested	[257]
	DLP (Digital Light Processing)	Vancomycin	GelMA with Zn-MOF	CELLINK Lumen XTM DLP bioprinter	-Osteomyelitis -Drug delivery system	-Compressive modulus 52.14 ± 19.42 kPa -If ZIF-8 nanoparticles are added, the compressive modulus increases to 128.13 ± 19.46 kPa -Enhanced cell adhesion, migration, and growth	-Sustained over 48 h -GelMA ~93% and GelMA/ZIF-8 ~88% cumulative	*Escherichia coli* DH5α; human adipose-derived stem cells (hASCs)	Not tested	[86]
		Deferoxamine (DPO) liposomes	TCP (tricalcium phosphate) + GelMA	AUTOCERA-M (Beijing, China)	-Bone defect -Drug delivery system	-GelMA microspheres diameter: 200 ± 30 µm -Porosity 64 ± 5% -Polydispersity index 0.19 ± 0.03 -Bioceramic compressive strength: 2.2 ± 0.2 MPa -Suitable for non-load-bearing applications	-Pure DFO liposomes: 67.6% in 6 h -Scaffold-loaded DFO: ~36% at 6 h; ~69% by 7 days -Total in vitro 48 h for pure liposomes	Rat MC3T3-E1	Rats divided into control, β-TCP, and TGL groups (N = 10 each; total N = 30).	[258]
	High-res Photopolymerization	Human parathyroid hormone (1-34 PTH)	XS-BIO resin; silver-coated syringe tip & steel counter-electrodes (electrodes as metals)	Miro P213 (Xingsheng)	-Osteoporosis -Drug delivery system	-No fracture in the microchannels at compressive loads of 40 N -No deformation for 20 s at forces of 20 N	Not specified	HUVECs	Female Sprague–Dawley rats (N = 30); OVX (N = 25) and sham (N = 5)	[259]
	Photo-crosslinking-assisted printing	Sorafenib	Methacrylated silk fibroin (SF-MA) + cetyltrimethylammonium bromide (CTAB); Ti_3_C_2_Tₓ MXene	Photo-crosslinking SF-MA gel setup	-Osteosarcoma model -Drug screening platform	-Controlled pore sizes -MXene increased the photothermal conversion -Laser irradiation was used to increase the scaffolds’ temperature and to favor the release of sorafenib	-pH- and laser-responsive -At pH 7: 76% at 5 h; pH 4.3: 18% at 5 h -Acidic + laser: 35% at 5 h (vs 18% without)	MC3T3-E1; MG-63	Not tested	[260]
Powder Bed Fusion (PBF)	SLS (Selective Laser Sintering)	Zoledronate	PLLA + mesoporous silicon (SBA15NH_2_)	Home-made SLS system	-Osteosarcoma -Drug delivery system	-In the acidic pH, the release is approximately two times higher, a key aspect for localized treatment of osteosarcoma or osteoporosis	-Initial burst phase release -Fast first 24 h, then slow -Acidic pH: 16.7% (24 h) + 21.7% later -Neutral pH: 9.6% (24 h) + 11.7% later	MG-63 (osteosarcoma); RAW264.7 (mouse macrophage/osteoclast precursor)	Not tested	[184]
	SLM (Selective Laser Melting)	Doxorubicin; indoleamine 2,3-dioxygenase inhibitor	Ti6Al4V + Zn(NO_3_)_2_·6H_2_O; Metal-organic-framework (MW) composite	Concept Laser M2	-Osteosarcoma -Drug delivery system	-High biocompatibility -Corrosion resistance -Bone-like elastic modulus -Microwave thermal sensitivity	-Acidic pH 5.5 + MW: 61.6% at 24 h; acidic no MW: 39.5% -Neutral pH 7.4: 27.2% (no MW) or 33.9% (with MW)	Murine OS K7M2; rBMSCs	BALB/c mice (male, 4–5 weeks) and male SD rats (numbers not specified)	[168]
		Strontium ranelate	Tannic Acid (TA) coated with PEG hydrogel	TRUMPF TruPrint 1000	-Osteoporosis model -Drug screening model	-The PEG coating increased the titanium crosslinkability -Optimal biodegradability -The release was diffusion-based, and it was slower than in other materials, given that the tannic acid scaffold is denser	-TA 20% → ~90% release in 2 days -TA 80% → ~50% release in 15 days	L929 mouse fibroblasts	Not tested	[261]
		Ethyl-2,5-Dihydroxibenzoate	Titanium coated with PLGA	Winforsys Metasys250	-Osteoporosis -Drug delivery system	-Inhibition of bone resorption -Promoter of bone formation -PLGA coating further stimulated the bone formation	Not specified	MC3T3-E1 mouse pre-osteoblasts	Female Sprague–Dawley rats (N = 20; 3 months old)	[262]
		Zoledronic acid (ZA)	JDBM Mg-Nd-Zn-Zr alloy + ceramic coating	ZRapid Tech SLM150	-Osteoporosis -Drug delivery systems	-Porosity: 80% -Pore-diameter: 600 µm -The ceramic coating reduced the biodegradability to ≈5 mg/day -Corrosion resistance	-Slow, controllable -~8% in first 3 days -~54% by 30 days (release driven by coating degradation)	Rat BMSCs (rBMSCs); mouse BMMs (bone-marrow macrophages)	Female Sprague–Dawley rats, 3-month-old (N = 60)	[185]
	EBM (Electron Beam Melting)	Paclitaxel	Titanium + PEG	Q10plus, GE, SUA	-Osteosarcoma -Drug delivery system	-In the acidic environment, the osteosarcoma cells were successfully killed	-pH-responsive: ~11% at 72 h (pH 7.4) -~85% at 72 h (pH 6.5) -Stable at 7.4, fast intracellular release at 6.5 -The releases were controlled for over 1 month	143B, HOS, MG63; SaOS2, U2OS	BALB/c nude mice (N = 30), 4 groups, n = 6/group	[263]
		Cisplatin	Ti6Al4V + PLGA-PEG-PLGA hydrogel	Arcam S12 (Molndal/Gothenburg, Sweden)	-Osteosarcoma -Drug delivery system	-Low elastic modulus -Low stiffness -Good osteoinduction -Large drug-loading due to the porous structure	-Initial burst then sustained >15 days -Release matched hydrogel degradation -Some hydrogel detachment observed	Osteosarcoma 143B, HOS, MG63	BALB/c nude mice, 5 groups (N = 5/group)	[162]
		Simvastatin	Porous Ti6Al4V (3DTi) + hydrogel	Arcam S12 (Molndal/Gothenburg, Sweden)	-Bone defect (osteosarcoma) -Drug delivery system -In vitro and in vivo (rabbit condyles)	-The tumor volume reduced by 59–77% without any other damage -The release was sustained for 18 days -Anti-osteosarcoma and osteogenic effects	-Continuous release up to 18 days aligned with hydrogel degradation	143B (human OS); L929 (mouse fibroblasts)	Six-week-old female BALB/c nude mice; 3 groups (n = 6 each).	[264]
Binder Jetting	Binder Jet 3D Printing	Curcumin loaded with Magnesium	β-TCP (β-tricalcium phosphate)	ExOne (Irwin, PA)	-Osteosarcoma -Drug delivery system	-In vivo osseointegration; -In vitro chemoprevention; -High cell viability;	-30 days: pH 7.4 ~22% (TCP) vs. ~17% (Mg-TCP); pH 5.0 ~30% (TCP) vs. ~23% (Mg-TCP) -Weibull fits R^2^ > 0.9	THP1-derived osteoclasts; hFOB; MG-63; S. aureus; P. aeruginosa	Sprague–Dawley rats (supplier Envigo, numbers not specified)	[203]
		Gingerol; Allicin	β-TCP	ExOne	-Osteoporosis -Drug delivery system	-Porosity: 50% -Antimicrobial activity -Ginger promoted osteoblast proliferation by over 50% -Reduced osteoclast activity -Porous core with dense exterior with 10 MPa compressive strength -Bone healing after 10 weeks	-Allicin sustained ~2 days (35–45%); gingerol release higher with PEG 50:50 vs. 65:35	Human fetal osteoblasts (hFOB); osteoclasts (THP1-derived)	Sprague–Dawley rats (N = 20)	[204]
		Quercetin; Vitamin D_3_ (lipid NPs)	β-TCP	ExOne	-Osteosarcoma -Drug delivery system	-Compressive strength around 10 MPa; -Osteoblast proliferation -Osteoclast inhibition -Antimicrobial activity against *Staphylococcus aureus* and *Pseudomonas aeruginosa*	-Biphasic; method- and pH-dependent; up to ~60 days -Power-law kinetics (R^2^ > 0.99) -↑ Acidic pH release -Above 80% release rate at pH 7.4 -Controlled release for 60 days	hFOB; THP-1-derived osteoclasts; MG-63; P. aeruginosa; S. aureus	Not tested	[197]
		Curcumin + Piperine (coated with Carvacrol)	β-TCP	ProMetal^®^ (ExOne)	-Osteosarcoma -Drug delivery system	-Compressive strength of 12.3 MPa -Pore diameter: 400 µm	-Biphasic; method- and pH dependent; -Up to ~60 days Power-law kinetics (R^2^ > 0.99) -↑ Acidic pH release -In combination with piperine, the curcumin availability increases to over 2000% -94% antibacterial activity -Cumulative release of 80% for curcumin and 90% piperine -Controlled release for 60 days	hFOB (passage 6); MG-63 (passage 6); *Staphylococcus aureus*	Not tested	[265]
		Gingerol	β-TCP	ExOne Innovent+	-Osteosarcoma -Drug delivery system	-Antioxidant properties -Tumor necrosis factor suppression -No cytotoxicity	-In a very acidic environment (pH 5.0), there is an initial burst release of 16% compared to 12% in pH 7.4	hFOB (ATCC CRL-11372); MG-63; *Staphylococcus aureus*	Not tested	[205]
		Retinol (vitamin A)	β-TCP scaffold with PCL/PEG coating	ExOne powder-bed printer	-Bone defect -Drug delivery scaffold	-Bulk density: ≈1.0 g/cm^−3^ -Porosity: 50% -Compressive strength: 5.1 ± 1.1 MPa	-Full release by 1 day at pH 5 -~50% plateau by 7 days at pH 7.4; Weibull fits reported;	hFOB; THP-1-derived osteoclasts	Not tested	[266]
		Vitamin D_3_	β-TCP scaffold with PCL/PEG coating	ExOne	-Bone defect -Drug delivery scaffold	-Pore diameter: 350 µm -Porosity: 48.9 ± 2.54% -Compressive strength: 7.0 ± 0.66 MPa -Relative density: 32.2 ± 1.23% -Osteoblast proliferation was enhanced by 64% compared to traditional osteoinductive methods	-3D scaffolds > porogen scaffolds -After 60 days in PBS: 3D-printed model released ~47.5% vs. porogen ~37.5%	hFOB	Not tested	[196]
		Sitafloxacin; Rifampin	α-TCP/HA; PLGA coating	Modified ZPrinter 450 (3D Systems)	-Osteomyelitis -Drug delivery system	-Reduced bacterial colonization -Improved mechanical robustness -The PLGA coating increased the Young’s modulus by three times, and the maximum stress by 4.4 times	->95% in 12 h (burst). PLGA-coated: biphasic -Initial burst then ~zero-order for 2 weeks (dose-dependent)	Not tested	Female Balb/cJ mice (13–15 weeks)	[76]

**Table 2:** Comparative summary table on 3D printing technology, release profile, and biocompatibility for each major drug class addressed in the manuscript.

Drug Class	Main 3D Printing Techniques	Release Profile	Biocompatibility/Biological Response	References
Antibiotics	FDM (PLA, PCL, PMMA) Bioprinting hydrogels (GelMA, methylcellulose/nHAp) DLP Binder Jetting (CaP); Melt-extrusion (PEOT/PBT) Micro-extrusion (SF-MA) MEW + FDM hybrid Melt-electrohydrodynamic PCL/PEG	Frequent early burst (often 30–95% within 6–48 h for some systems), then sustained release typically to ~30 days Some systems longer (e.g., ciprofloxacin in PEOT/PBT sustained ≥1 month) MOF/hydrogel could potentially increase release profiles	Overall biocompatible carriers (PLA/PCL/GelMA/CaP/Ti) Strong antimicrobial activity, reduced colonization, preserved cell viability, and bone integration PMMA curing can generate heat High local rifampin can be cytotoxic if overdosed (not at reported loads)	[76,86,94,106,109,118,134,150,153,248]
Anti-inflammatory	FDM (PLA) Bioprinting (PCL/alginate/gelatin with drug microparticles) Modified/custom extrusion (PCL + liposomes)	Surface-loaded parts can show fast burst (e.g., ~50% dexamethasone in 6 h), while embedded/encapsulated systems show tempered burst and sustained release ~2–4 weeks (e.g., DEX MPs to day 30)	Cytocompatible Osteoinductivity reported for some constructs Degradation stable and tunable via composite design	[194,239,248,256]
Anticancer	SLA/DLP SLS/SLM/EBM (Ti alloys and ceramics) Binder Jetting (β-TCP) Photo-crosslinking-assisted printing Tumor-on-chip/microfluidic platforms	Highly tunable: from ultra-fast (e.g., 5-FU complete in ~2 h) to prolonged/pH- or stimulus-responsive (e.g., paclitaxel >1 month at pH 7.4; faster at pH 6.5; cisplatin sustained >15 days; DOX release boosted by microwave/acidic pH) Overall span ≈ 2 h to ≥60 days depending on drug/carrier	Effective local tumor suppression with minimal systemic exposure Maintained osteogenesis/osseointegration of scaffolds Responsive systems (pH/thermal/photothermal) allow on-demand dosing	[162,168,184,185,233,240,243,252,259,263]
Other drugs and growth factors	FDM Bioprinting (alginate/gelatin, PVA, GelMA) SLS/SLM Binder Jetting MEW hybrid	Wide spectrum: from fast burst (e.g., VEGF, leonurine early 72 h burst) to very prolonged: BMP-2 (sustained 2+ weeks; ZIF-8 composite ~57% by 174 h and continuing), Salvianolic acid B (~60% by day 80), vitamins/natural products like Vitamin D_3_ and Gingerol sustained up to 60 days, Dipyridamole sustained 30 days (no burst)	Strong osteogenesis and angiogenesis Enhanced osteoblast proliferation and osseointegration Osteoclast inhibition (e.g., Strontium ranelate); generally biocompatible with occasional initial bursts governed by coating/encapsulation	[196,197,199,200,204,205,249,259,261,265,266]

## Data Availability

No new data were created or analyzed in this study.
